# Estimating the genetic parameters of resilience toward known and unknown disturbances in sheep using wool fibre diameter and body weight variability

**DOI:** 10.1186/s12711-025-00983-1

**Published:** 2025-07-14

**Authors:** Erin G. Smith, Samuel F. Walkom, Dominic L. Waters, Sam A. Clark

**Affiliations:** 1 School of Environmental and Rural Science, University of New England, Armidale, NSW 2351 Australia; 2 Animal Genetics and Breeding Unit, University of New England, Armidale, NSW 2351 Australia

## Abstract

**Background:**

General resilience in animals can be quantified by analysing the variability in longitudinal data. However, it is unclear whether resilience indicators derived from different longitudinal data series can predict resilience to known or unknown disturbances in sheep. This study aimed to use two sources of longitudinal data, wool fibre diameter and body weight, to develop potential indicators for resilience to the known stress of weaning and overall resilience to unknown disturbances. The genetic parameters of these traits were assessed, along with the genetic correlations between traits from different data series and different definitions of resilience. Additionally, correlations between resilience indicators, health and production traits were estimated to evaluate the suitability of including resilience indicators in breeding programs.

**Results:**

Fibre diameter and body weight records from approximately 6500 yearling Merino sheep were used to estimate four resilience indicators of resilience towards unknown disturbances: log-transformed variance (Lnvar), lag-1 Auto (Auto), skewness (Skewness) and absolute difference in the deviations (ABS) from these curves. Three other traits, rate of change in the response and recovery (ROC_response and ROC_recovery) and area between curves (ABC) during a known disturbance of weaning, were also estimated. Resilience indicators were found to be lowly heritable (0.03 ± 0.01 to 0.18 ± 0.04). Genetic correlations between the general resilience indicator and the indicator of resilience to weaning stress were generally moderate, particularly in the wool fibre diameter data, suggesting these may represent similar traits. Genetic correlations between resilience indicators derived from wool fibre diameter and body weight data were typically weak to moderate, which indicates that they possibly capture different aspects of resilience. The genetic correlations between resilience indicators and health traits were mostly low, except for body condition score. Correlations between resilience and production traits were low to moderate and favourable.

**Conclusions:**

Resilience indicators based on deviations in wool fibre diameter and body weight can be used to potentially select animals that are less affected by environmental disturbances. The genetic correlations between resilience indicators and health and production traits suggest that these traits could be included in breeding programs to improve resilience without adversely affecting production traits.

**Supplementary Information:**

The online version contains supplementary material available at 10.1186/s12711-025-00983-1.

## Background

Assessing the resilience of livestock toward environmental disturbances is crucial to the optimisation of future breeding programs to enhance the capacity of animals to cope and adapt to changing environments. Longitudinal data have featured prominently in the development of resilience indicators over the last decade, owing to their ability to record short-term variation in productive output [1, 2]. When an animal is affected by a disturbance in its internal or external environment, the supply of metabolic resources to support anabolic activities associated with growth, production and reproduction is altered. These deviations have served in the formation of several trait definitions of resilience, which have a genetic component and are favourably correlated with greater health, welfare and productive outputs [3, 4]. Resilient animals are expected to exhibit more consistent production over time, with fewer and smaller deviations, as they are less affected by disturbances compared to less resilient animals [5]. However, many resilience studies only examine a single biological parameter to extract resilience phenotypes (e.g., egg production, milk yield, growth), despite several authors emphasising the multi-faceted nature of resilience [6, 7].

The frequency of data recording, the sensitivity of the tissue structure to anabolic and catabolic signals and the integration of metabolic pathways are all factors that could influence the type of resilience examined. Abdelkrim et al. [8] examined deviations in milk yield and body weight and found that only 20% of perturbations were synchronised between the two data sources. Gorssen et al. [9] similarly reported that resilience indicators based on deviations in body weight, feed intake and feeding behaviours were substantially different from one. These findings highlight the diverse responses that can be captured from different data series. Colditz et al. [7] suggest that multiple body functions measured at different resolutions may need to be considered to attain a comprehensive understanding of resilience to disturbances of different origins (physical and psychological) and on different time scales. However, a limited number of genetic evaluations have attempted to determine the correlations between trait definitions of resilience between data types and disturbances of both known and unknown origins.

Current assessments of resilience typically only examine resilience toward disturbances of either known origin or unknown origin, not concurrently. It is therefore unclear whether their resilience towards specific disturbances is sufficiently correlated to resilience toward unknown challenges when using longitudinal data. Assessing responses to unknown priors relies on the assumption that animals are constantly subject to unknown disturbances, which result in fluctuations in frequently recorded traits (black box approaches) [4]. In contrast, assessing animal responses to known disturbances may provide a more accurate descriptor of the diversity of individual responses to a challenge for selection purposes [10, 11], but is less frequently conducted due to the constraints of experimentally induced disturbances. Berghof et al. [12] suggest that any information regarding the nature of the disturbances that occurred would be valuable for further defining and validating the resilience indicators derived from longitudinal data.

Routine management practices, such as weaning, can be used as a known disturbance. In sheep production systems, weaning can be a critical phase of production and is often characterised by changes in diet and social and environmental conditions [13]. Poor adaptation to the stress of the weaning event can often result in a condition known as weaner ill thrift syndrome, which is characterised by rapid weight loss, reduced immune function, loss of production and high mortality among young sheep [14].

Wool fibre diameter, measured along the length of the wool staple (a bundle of wool fibres), offers a unique source of longitudinal data for assessing resilience in sheep. Wool fibres grow continuously throughout the year, even under severe nutritional stress, making them a valuable trait for studying resilience [15]. The growth of wool fibre diameter is sensitive to various environmental and physiological factors, including nutrition, diseases, parasites, climatic conditions, and reproduction [16–20]. While wool length growth can also be influenced by these factors, recording its variation on a large scale is more challenging, typically requiring skin dye bands or isotope analysis. The variation in wool length and fibre diameter growth rates across the year occurs non-linearly between animals along the staple length [21]. This can make using longitudinal wool fibre diameter to measure resilience to known stress events, such as weaning, more complex. Statistical methods exist that can separate amplitude variation from phase variation in longitudinal data, with phase variation reflecting differences in the timing and speed of growth events [22]. Explicitly modelling phase variability could aid in distinguishing the animal’s responses to known challenge events.

The objective of this study was to use two sources of longitudinal data found in sheep (fibre diameter and body weight) to extract traits describing response and recovery from both known (weaning) and unknown disturbance events. The genetic parameters of these traits were used to assess the correlations between resilience traits from different data sources and disturbance events. To validate the usefulness of these potential resilience indicators, the genetic and phenotypic correlations with health and production were estimated. It was hypothesised that the variability in wool fibre diameter and body weight could provide a more comprehensive overview of how animals respond to disturbances in their environment.

## Methods

The analyses in this paper were separated to accomplish three main objectives. First, the longitudinal records of fibre diameter and body weight data were processed into smooth functions and temporally aligned to remove phase variation. Second, trait definitions of resilience were developed based on the overall variability in the data and the specific response and recovery to weaning disturbance. The heritabilities and genetic correlation of all resilience indicators were estimated to determine if there are differences between resilience trait definitions and data types, which could be used to select animals that are potentially more resilient to environmental disturbances. Third, genetic correlations between the resilience, health and production traits were estimated.

### Dataset

The dataset used in this study contained longitudinal fibre diameter of wool and body weight records of a cohort of around 6500 Merino and Poll Merino yearlings. The records were collected over 8 years between 2007 and 2014 in the Sheep Cooperative Research Centre Information Nucleus Flock. This flock was composed of eight flocks located across southern Australia. The data set contains 41 flock-year groups with an average size of 150.9 animals (min 49, max 386: more information can be found in [23] and [24]). A comprehensive description of the design and purpose of the Information Nucleus Flocks was provided by [25].

At approximately 10 months of age (min 7, max 13 months), a wool staple was taken from the animal and measured for fibre diameter at 5mm increments along the length of the wool staple using Optical Fibre Diameter Analyser 2000 instrumentation (OFDA2000) [26]. Further details of this process have previously been described by [24]. This technology produces a series of fibre diameter measurements along the wool staple length for each animal. The period of wool growth corresponds to the time period between the animal's birth and the sampling date. Each animal had an average of 16.6 fibre diameter records (min 10 records and max 27 records) depending on the length of the wool staple. These animals also had frequently collected body weight measurements between birth and the date of wool sampling, with an average of 13 body weight measurements throughout this time (min 5 and max 20). Wool fibre diameter and body weight records were standardised to a mean of zero and a standard deviation of one across individuals within their respective flock-year group and recording moment for body weight, to correct for scaling effects and known flock-year differences in trait performance consistent with [27]. The final dataset contained 106,396 fibre diameter and 80,301 bodyweight records.

In the latter part of this study, genetic and phenotypic correlations were estimated between resilience indicators and six commonly reported health traits in sheep. This analysis aimed to determine whether resilience indicators reflect specific measures of resilience and resistance to major internal and external parasitic diseases and bacterial infections, with the expectation that more resilient animals are healthier than less resilient ones. These health indicator traits included dag score (DAG) and breech wrinkle score (BRWR) which are indicators of susceptibility to flystrike and internal parasites; fleece rot score (FLROT) and wool dermatophilosis score (DERM) which are bacterial infections of the skin; worm egg count (WEC), which is an indicator of parasite resistance and resilience and body condition score (CS), an indicator of overall health. Definitions are provided in detail by [28]. The traits CS and WEC were assessed between 120 and 210 days of age, while the remaining traits were assessed between 210 and 300 days of age. The trait WEC was transformed using a cube root transformation before the analysis due to the right-skewed distribution of the raw data.

The genetic and phenotypic correlations between resilience indicators and eight major production traits currently included in the genetic evaluation of Australian Merino sheep were estimated. These traits included clean fleece weight (CFW), mean fibre diameter (MFD), fibre diameter coefficient of variation (FDCV), staple strength (SS), staple length (SL), weaning weight (WT: 120–210 days of age), scanned eye muscle depth (EMD) and scanned fat depth at the 12th rib (FAT). Further descriptions of these traits can be found in [29]. The fleece traits (CFW, MFD, FDCV, SS and SL) were assessed between 300 and 400 days of age, while the scanned carcase traits (EMD and FAT) were evaluated between 120 and 210 days of age. Animals without resilience phenotypes but with health and production records and born in the same flock year, and linked by common sires, were included in this part of the analysis to increase the number of trait records (3672 additional animals). Outliers for each of the traits were removed where the trait value exceeded 3SD from the mean of the flock year group.

### Converting fibre diameter and weight records into functional representations

To convert the wool fibre diameter and body weight measurements into a continuous representation, the approach termed Functional Data Analysis by Ramsay and Silverman [30] was adopted. Functional data analysis is a framework for turning cross-sectional measurements into continuous functions. The objective function $\left[{f}_{perf}\left(\mathbf{c}\right)\right]$, (where ${f}_{perf}$ is either fibre diameter or body weight) being minimised takes the general form:

$${f}_{perf}\left(c\right)= \sum_{j=1}^{n}{[{y}_{j }-x\left({t}_{j }\right)]}^{2}+\lambda {\int }_{0}^{L}{[{\partial }^{2}x\left(\mathbf{c},\boldsymbol{ }t\right)]}^{2} \text{d}t,$$

1 $$x\left(\mathbf{c},t\right)=\mathbf{c}{\prime}\varphi \left(t\right),$$

where, ${y}_{j}$ represents $n$ observed values of fibre diameter or body weight of an animal $j$ measured on the interval (days or staple length) $[0, L]$. The term $x\left(\mathbf{c},t\right)$ is the smooth function constructed from a set of B-spline basis functions $\varphi \left(t\right)$ which were linearly combined with coefficients vector $\mathbf{c}.$ B-spline basis functions were selected due to their flexibility, smoothness and localised control enabled using knot points. The notation ${\partial }^{2}x\left(\mathbf{c},\boldsymbol{ }t\right)$ refers to the second derivative of $x\left(\mathbf{c},t\right)$ with respect to staple length or time $\left(t\right)$ and defined by its differential $\left(\text{d}t\right)$. The scalar $\lambda$ controls the relative emphasis given to the goodness of fit compared to smoothness. When $\lambda$ is low, $x\left(\mathbf{c},t\right)$ adjusts to the data as well as possible reducing the squared error. With larger values of $\lambda$ more weight is placed on the penalty term causing the second derivative of the function to evolve towards a straight line (curvature of zero).

The transformation of the fibre diameter and weight data into smooth functions was performed in R using the package ‘*fda*’ [31]. The basis functions were constructed using the command ’create.bslpine.basis’ and the order of the b-spline was specified by the argument ‘norder’ which has $n-1$ degree. The value of ‘norder’ was set to three and four, respectively for the body weight and fibre diameter data, based on preliminary testing (not shown). The ‘Lfdobj’ term which was used to define the smoothness of the first and subsequent derivatives of the function, was set to two for both body weight and fibre diameter, meaning the second derivatives are penalised. However, the second derivative curves were not investigated in this study due to the complexity of their biological interpretation. For the fibre diameter data, the knot points or ‘breaks’ were specified based on 10 quantiles defined within the flock-year group. A similar approach was taken with the body weight data using 10 quantiles and additional specified knot points located at sparse areas of the data, which occurred between (0 and 90 days). This sparse area of data occurred because the lambs were not weighed between birth and weaning. A value of $\lambda =2500$ was used to capture the short-term variation in fibre diameter and body weight of each animal. This value was chosen based on a visual inspection of the fibre diameter and body weight curves reflecting the inherent shape of the curves and prior knowledge from the literature [8, 10, 32]. The choice of time step for prediction was set at 1mm increments (3 days) of staple length for fibre diameter and 10-day intervals for body weight. This enables the retrieval of predicted trait response values at a higher frequency than those observed and at consistent intervals, as in the case of the body weight data. These prediction intervals were chosen to ensure the trait responses still behaved in accordance with the expected physiology of the trait. Once the functions were estimated, the results were stored for further analysis.

### Temporal alignment of fibre diameter and body weight functional data

Before the trait definition of resilience could be determined, the fibre diameter and body weight data were further processed to remove phase variation. Unaccounted phase variation can obscure resilience traits, especially in response and recovery to a specific disturbance. In wool fibre diameter, this variation arises from differences in wool growth rates, staple length at birth and OFDA2000 measurement errors. For body weight, it stems from age differences among animals. Simple methods such as linear time-scaling do not work well, particularly on wool fibre diameter, because they rely on overly simplistic assumptions such as uniform rates of change and consistent timing of key events across all samples, which do not apply. Instead, non-linear methods can be explored to align the data accurately and define resilience traits effectively.

Phase variation can be addressed using temporal alignment (or time warping), which synchronises features by adjusting the time axis. This involves transforming the time scale of each function to minimise phase differences while preserving the data's shape. Techniques for temporal alignment include dynamic time warping, landmark regression and elastic functions like the Square Root Velocity Function (SRVF: reviewed by [22]). Among these, elastic functions are increasingly recognised as effective across various applications, such as activity data and image analysis [33].

The SRVF is a pre-processing method that takes a time series and removes some rate variations. Unlike methods like dynamic time warping or landmark registration, SRVF is superior due to its use of Riemannian geometry, which measures distance on a curved shape [34]. This approach captures not only superficial differences but also the underlying structure and patterns between curves, offering a more accurate comparison. Other methods can suffer from the "pinching effect", forcing misaligned curves into sync, which can lead to misleading results [33]. SRVF, however, respects the data's natural shape and structure, preserving phase and amplitude relations for more meaningful analysis [35].

In the following section, the temporal alignment of fibre diameter data is described using the SRVF transformation developed by and implemented using the R package ‘*fdasrvf*’ [36]. Here, a general description of the algorithms is provided; however, a comprehensive statistical background can be found in [37] and [34]. The alignment of individual animal functions occurred within their flock-year group to ensure that the overall pattern of the fibre diameter and growth curve was preserved.

#### Convert function to SRVFs

The SRVF representations of a given animal's fibre diameter and body weight functions were defined as;

2 $${q}_{j}\left(t\right)= \text{sign}\left({\dot{f}}_{j}\left(t\right)\right)\sqrt{\left|\dot{{f}_{j}} \left(t\right)\right|},$$

where $\dot{{f}_{j}}(t)$ was the derivative of the original fibre diameter or body weight function ${f}_{j}$ with respect to original staple length or time $(t).$ This derivative measures the rate of change at each staple length/time point. The absolute derivative captures the intensity of the change while the square root stabilises variances across different sections of ${f}_{j},$ which aids the comparison between functions with different magnitudes but similar structures. The sign function indicates whether ${f}_{j}(t)$ is increasing or decreasing, preserving both direction and scale for further analysis.

#### Fréchet mean curve of the SRVFs belonging to each flock year group

The next step was to calculate the Fréchet mean (or Karcher mean) curve for each flock year. The Fréchet mean provides a central tendency measure for a set of curves in a geometric space, which aims to identify an average curve that minimises the variance within a set of aligned curves for a given flock year. For flock year $k$ the Fréchet mean ${u}_{k}$, represented as SRVFs, is the function that minimises the sum of squared differences to all other curves in the set. It is expressed as:

3 $${u}_{k}=\text{arg}{min}_{uk} {\sum }_{j=1}^{N}{d({\mu }_{k}, {q}_{jk})}^{2},$$

where ${q}_{jk}$ are the SRVF representations of the aligned individual functions from flock year $k$, $d$ represents the Riemannian distance metric which quantifies the difference between two SRVFs, and $N$ is the number of functions in flock year $k$. The Fréchet means provides a statistically robust measure of central tendency, which is crucial for analysing variations around common patterns in the data. Later, the Fréchet mean curve of each flock year group was also utilised to calculate the deviation between the animal's response curve and their flock year groups Fréchet mean.

#### Optimisation of the warping function for each function

Using the Fréchet mean curves, the warping function for each individual animals fibre diameter and body weight function was optimised. The objective was to find the optimal warping function (${{\gamma }_{j}}^{*})$ that aligns each individual function to the Fréchet mean curve of their flock-year. Each ${{\gamma }_{j}}^{*}$ represents the temporal deformation needed to align the animal function with the central trend represented by the Fréchet mean. The optimal warping function ${{\gamma }_{j}}^{*}$ is obtained by solving the following optimisation problem;

4 $${{\gamma }_{j}}^{*}={\mathit{argmin}}_{\gamma \in\Gamma }\text{ (}\Vert {\mu }_{k}-\left({q}_{j} ^\circ \gamma \right) \sqrt{\dot{\gamma }}\Vert +\lambda R(\gamma )).$$

The search for $\gamma$ is conducted over the set Γ called the warping group. The warping group is a set of all possible warping functions that meet the boundary conditions of the unit interval [0, L]. These functions are diffeomorphisms of the unit interval [0, L], meaning they are smooth, invertible functions with smooth inverses that map the interval onto itself preserving the endpoints. The regularisation term $R(\gamma )$ penalises excessive warping controlled by the parameter $\lambda$.

The degree of alignment between curves depends on the value of lambda (distinct from the lambda used for smoothing). A lambda of 0 allows complete flexibility, enabling greater distortion, whereas a larger lambda enforces stricter alignment, making the transformation closer to the identity function. Selecting an appropriate lambda is crucial to balancing alignment accuracy and preserving biological variability. For both fibre diameter and body weight functions, four lambda values (0, 0.2, 0.4 and 0.6) were tested to assess how alignment flexibility influences resilience trait definitions. These values, chosen based on preliminary testing, represent a spectrum from highly flexible to more rigid alignments. The genetic and phenotypic correlation was calculated to assess the impact of these lambda values on trait definitions.

The final step in the temporal alignment was to apply the optimal warping function by composition to the original function ${f}_{j}$ (fibre diameter or body weight). This effectively transforms ${f}_{j}$ to align it temporally with the Fréchet mean curve and is shown as;

5 $${\dot{f}}_{j}\left(t\right)={f}_{j}\circ {{\gamma }_{j}}^{*}.$$

Together, the warping function optimisation is solved with dynamic programming to ensure the solution is computationally efficient and finds the best alignment. Once the alignment was complete, the animal's original, aligned and warping functions were extracted along with the Fréchet mean curve for each flock year.

### Calculation of resilience indicators

Two categories of resilience indicators were created from the aligned fibre diameter and body weight functions. The first were traits suggested to capture the resilience of the animals toward unknown disturbances. The second group of traits were created by identifying resilience specifically toward a known disturbance, in this case, weaning.

#### Resilience to unknown disturbances

The deviations of an individual’s fibre diameter and body weight function from the Fréchet mean curve of the flock-year group were used to calculate resilience parameters. The deviations were calculated as the observed performance of the individual minus the performance of the flock-year group, which aims to assess the animal's performance in the face of disturbances relative to the flock-year group. The deviations were used to calculate four traits that are expected to capture the animal’s overall resilience: natural logarithm of the variance (Lnvar), the lag^−1^ autocorrelation of the deviation (Auto), skewness of the deviations (Skewness) and the mean absolute change in the deviations (ABS). The traits were calculated in both fibre diameter (FD) and body weight (BW) data.

The Lnvar was calculated as;

6 $${Lnvar}_{j}= ln\left(\frac{{\sum }_{i=1}^{{n}_{j}}({x}_{ij}-{\overline{x} }_{j}{)}^{2}}{{n}_{j}-1}\right),$$

where ${x}_{ij}$ was the deviation $i$ of the animal $j$, ${\overline{x} }_{j}$ is the mean of the deviation of the $j$ individual, ${n}_{j}$ is the number of deviation of the $j$ animal and $ln$ is the natural logarithm. Animals that have lower values closer to zero are suggested to be more resilient (less variation being less affected by environmental disturbances) compared to higher values, indicating more deviations and hence lower resilience. The variance was transformed using a logarithmic scale to correct the right-skewed distribution typical of variance phenotypes.

The autocorrelation of (Auto) of the deviation was calculated as;

7 $${Auto}_{j}= \frac{\sum_{i=1}^{{n}_{j}-1}({x}_{ij}- {\overline{x} }_{j})({x}_{\left(i+1\right)j}- {\overline{x} }_{j})}{{\sum }_{i=1}^{{n}_{j}}({x}_{ij}- {\overline{x} }_{j}{)}^{2}},$$

where ${n}_{j}$ was the number of pairs of subsequent deviation of animal $j$, ${x}_{ij}$ was the deviation $i$ of the $j$ animal, ${\overline{x} }_{j}$ is the mean of the deviation of animal $j$ and ${x}_{(i+1)j}$ was the deviation this was immediately subsequent to ${x}_{ij}$ animal $j.$ The autocorrelation measures the degree to which deviations are correlated with their subsequent deviations and indicates the duration of environmental disturbances. A low Auto of the deviation is suggested to indicate greater resilience because resilient animals have fewer and shorter periods of negative deviations compared to less resilient animals. Positive Auto values indicate that deviations are persistent in the same direction. If an individual experiences a deviation, subsequent deviation tends to be in the same direction. This implies a slower recovery process as the effect of the disturbance lingers over time. A slower recovery may be generally less desirable as it indicates that the individual remains affected by the disturbance for a longer period.

The skewness of the deviation provides information about the direction and intensity of environmental disturbances and was calculated as;

8 $${skewness}_{j}= \frac{{\sum }_{i=1}^{{n}_{j}}\left(\frac{{x}_{ij}-{\overline{x} }_{j}}{{s}_{j}}\right)}{{n}_{j}},$$

where ${s}_{j}$ is the standard deviation of the deviations of the animal $j$ calculated as;

9 $${s}_{j}= \sqrt{\frac{1}{{n}_{j}-1} \sum_{i=1}^{{n}_{j}}({x}_{ij}-{\overline{x} }_{j}{)}^{2}}.$$

A skewness of zero suggests minimal environmental influence whereas positive skewness indicates a favourable response to an environmental factor and a negative skewness signifies a negative impact of the environmental disturbance on the animal.

The mean absolute difference of deviation (ABS) represents the average absolute difference in the deviation over the time period and was calculated as;

10 $$ABS= \frac{1}{n-1} {\sum }_{i=2}^{n}| {x}_{i }- {x}_{i-1}|.$$

Higher ABS values indicate that there are larger changes in the deviation between staple length or time points. This could suggest more variability of fluctuations in the deviation over time. Lower ABS values indicate smaller changes in the deviation values from one time point to the next, suggesting more stability or less fluctuation in the deviation values over time.

#### Calculating resilience indicators to the known disturbance of weaning

A second set of traits was created to assess the animal's response to weaning, which occurred at an average age of 90 days (min 62, max 119). Body weight records at weaning were used to approximate the weaning period in the aligned body weight data. For fibre diameter, the weaning period was estimated by scaling the corresponding ages from the body weight data proportionally to the staple length.

The weaning disturbance period was identified at the flock-year level using the Fréchet mean curve and consisted of two phases. The first was the collapse phase, marked by a decline in trait value as the animal reacted to weaning. The second was the recovery phase, characterised by an upward trend in trait performance. This framework aligns with study definitions of resilience toward known disturbances [1, 38]. In 24 flock-year groups, no clear weaning challenge was detected, so they were excluded from this part of the analysis.

The trait definitions created from this region of the data attempted to summarise the magnitude of the response and recovery from a specific disturbance of the weaning. The trait definitions include the rate of change in the response phase (ROC_response), the rate of change in the recovery phase (ROC_recovery) and the area between the individual's observed performance curve and the performance curve of the flock year (ABC). For each resilience indicator, animals with trait values more than 3SD from the mean of their respective flock-year group were considered outliers and discarded. All calculations of the resilience indicators and plotting were performed in R 4.2.3 software [39].

**Fig. 1:**
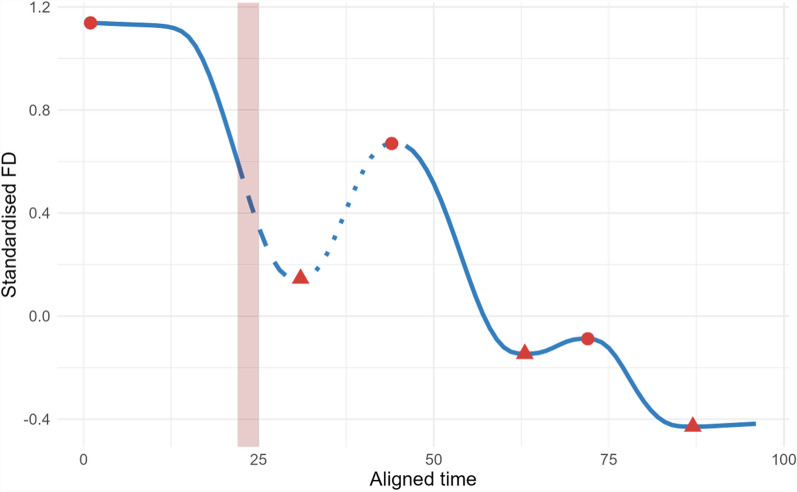
Example of an individual animal showing the estimated weaning period (indicated by shading) on an aligned fibre diameter curve. The estimated response phase to the possible weaning disturbance (dashed line), the recovery phase (dotted line), localised minimums (triangles) and maximums (circles)

The rate of change in the response phase was calculated as the change in the performance between the start of weaning to the next local minimum, whereas the rate of change in the recovery phase was calculated as the difference between the local minimum and the next local maximum of the flock year curve. The local minimum and maximum were identified in the fibre diameter and weight curves using a process-driven function similar to Borchers [40]. An example of this process is given in Fig. 1.

The area between curves (ABC) during the weaning disturbance was calculated using the ‘MESS’ [41] R package using the ‘auc’ command to calculate individually the area under the curves for the animal's observed curves and the flock-year curves before calculating the difference between the two curves. Animals with negative ABC values suggest that their trait performance is less than the flock-year response to weaning, which may suggest that these animals had greater responses and slower recovery from disturbance. Values of ABC that are positive or close to zero had trait values greater or similar to the flock-year, which are suggested to be more resilient to the weaning disturbance.

### Genetic parameters

Variance components and heritabilities were estimated using univariate single-step animal models. The genetic and phenotypic correlations between different resilience indicators and between resilience indicators, health and production traits were estimated using bivariate single-step animal models. The analyses were performed with BLUPF90 + programs [42] with estimates conducted using REML procedures. The pedigree consisted of 18,5629 animals from three generations. Genomic information (59,208 single nucleotide polymorphisms (SNPs)) was available for 6688 animals in total, of which 4143 had resilience phenotypes. The imputation of the genomic data in this flock was previously described by Daetwyler et al. [43]. Genomic information was included in the univariate and bivariate models using matrix $\mathbf{H}$ [44], which combined the pedigree ($\mathbf{A}$) and genomic ($\mathbf{G}$) relationship matrices (constructed using VanRaden [45]). The two matrices were combined to form the $\mathbf{H}$ matrix using an alpha value of 0.95.

### Univariate analysis

Variance components and heritabilities were estimated using the following univariate linear mixed animal model;

11 $$\mathbf{y}=\mathbf{X}\mathbf{b}+{\mathbf{Z}}_{1}\mathbf{a}+{\mathbf{Z}}_{2}\mathbf{m}+\mathbf{e},$$

where $\mathbf{y}$ was a vector with phenotypes for the resilience indicator; $\mathbf{b}$ was a vector of fixed effects which included birth rearing type (3 levels, born single reared single, born mutltiple reared single and born multiple reared multiple) and contemporary group (76 levels, a combination of flock-year-sex); $\mathbf{a}$ was a vector containing the additive genetic effects, which was assumed to follow a normal distribution for the $\mathbf{H}$ matrix combining both pedigree and genomic relationship matrices using a single step genomic evaluation $\mathbf{a}=N\left(0, \mathbf{H}{\upsigma }_{\text{a}}^{2}\right),$ where ${\upsigma }_{\text{a}}^{2}$ was the additive genetic variance; $\mathbf{m}$ was a vector of maternal permanent environmental effects, assumed to follow $\mathbf{m}=N\left(0,\mathbf{I}{\upsigma }_{\text{m}}^{2}\right)$ where $\mathbf{I}$ was the identity matrix and ${\upsigma }_{\text{m}}^{2}$ was the maternal variance; $\mathbf{e}$ was the vector of residual effects, assumed to follow the a normal distribution $\mathbf{e}=N\left(0,\mathbf{I}{\upsigma }_{\text{e}}^{2}\right)$ where ${\upsigma }_{\text{e}}^{2}$ was the residual variance. $\mathbf{X}$ and ${\mathbf{Z}}_{1}$ and ${\mathbf{Z}}_{2}$ are incidence matrices that link the records in $\mathbf{y}$ to the fixed, additive and maternal permanent environmental effects, respectively.

#### Bivariate analysis

Bivariate animal models were used to calculate the genetic and phenotypic correlations between resilience indicator traits, between resilience indicators and health traits and between resilience indicators and production traits. The model applied for the bivariate analysis between each pair of traits of took the general form;

12 $$\left[\begin{array}{c}{\mathbf{y}}_{1}\\ {\mathbf{y}}_{2}\end{array}\right]=\left[\begin{array}{cc}{\mathbf{X}}_{1}& 0\\ 0& {\mathbf{X}}_{2}\end{array}\right]\left[\begin{array}{c}{\mathbf{b}}_{1}\\ {\mathbf{b}}_{2}\end{array}\right]+\left[\begin{array}{cc}{\mathbf{Z}}_{\mathbf{a}_1}& 0\\ 0& {\mathbf{Z}}_{\mathbf{a}_2}\end{array}\right]\left[\begin{array}{c}{\mathbf{a}}_{1}\\ {\mathbf{a}}_{2}\end{array}\right]+ \left[\begin{array}{cc}{\mathbf{Z}}_{\mathbf{m}_1}& 0\\ 0& {\mathbf{Z}}_{\mathbf{m}_2}\end{array}\right]\left[\begin{array}{c}{\mathbf{m}}_{1}\\ {\mathbf{m}}_{2}\end{array}\right]+\left[\begin{array}{c}{\mathbf{e}}_{1}\\ {\mathbf{e}}_{2}\end{array}\right],$$

where ${\mathbf{y}}_{1}$ and ${\mathbf{y}}_{2}$ are the vectors with phenotypes for the first and second traits; ${\mathbf{b}}_{1},{\mathbf{b}}_{2}$**,** ${\mathbf{a}}_{1},{\mathbf{a}}_{2},{\mathbf{m}}_{1},{\mathbf{m}}_{2},{\mathbf{e}}_{1}$ and ${\mathbf{e}}_{2}$ are the vectors containing the fixed effects, additive, maternal permanent environmental and residual effects for traits 1 and 2, respectively;${\mathbf{X}}_{1},{\mathbf{X}}_{2},{\mathbf{Z}}_{\mathbf{a}_1}$**, **${\mathbf{Z}}_{\mathbf{a}_2},{\mathbf{Z}}_{\mathbf{m}_1}$ and ${\mathbf{Z}}_{\mathbf{m}_2}$ are incidence matrices associating observations to fixed, additive and maternal permanent environmental effects for traits 1 and 2, respectively. The significant fixed effects for these traits included birth-rearing type and contemporary group. Body weight recorded at the time of assessment was fitted as a covariate for the scanned traits EMD and FAT. The random effects were assumed to be normally distributed with a mean of zero and a (co)variance structure equal to:

$$\left[\begin{array}{c}{\mathbf{a}}_{1}\\ {\mathbf{a}}_{2}\end{array}\right] \sim N\left(\left[\begin{array}{c}0\\ 0\end{array}\right], \mathbf{H}\otimes \left[\begin{array}{cc}{\upsigma }_{\text{a}_1}^{2}& {\upsigma }_{\text{a}_1\text{a}_2}\\ {\upsigma }_{\text{a}_1\text{a}_2}& {\upsigma }_{\text{a}_2}^{2}\end{array}\right]\right),$$

$$\left[\begin{array}{c}{\mathbf{m}}_{1}\\ {\mathbf{m}}_{2}\end{array}\right] \sim N\left(\left[\begin{array}{c}0\\ 0\end{array}\right], \mathbf{I}\otimes \left[\begin{array}{cc}{\upsigma }_{\text{m}_1}^{2}& {\upsigma }_{\text{m}_1\text{m}_2}\\ {\upsigma }_{\text{m}_1\text{a}_2}& {\upsigma }_{\text{m}_2}^{2}\end{array}\right] \right),$$

and

$$\left[\begin{array}{c}{\mathbf{e}}_{1}\\ {\mathbf{e}}_{2}\end{array}\right] \sim N\left(\left[\begin{array}{c}0\\ 0\end{array}\right], \mathbf{I}\otimes \left[\begin{array}{cc}{\upsigma }_{\text{e}_1}^{2}& {\upsigma }_{\text{e}_1\text{e}_2}\\ {\upsigma }_{\text{e}_1\text{e}_2}& {\upsigma }_{\text{e}_2}^{2}\end{array}\right]\right),$$

where $\mathbf{H}$ is single-step genomic relationship matrix;$\mathbf{I}$ was an identity matrix;${\upsigma }_{\text{a}_1}^{2}, {\upsigma }_{\text{a}_2}^{2}$, ${\upsigma }_{\text{m}_1}^{2}, {\upsigma }_{\text{m}_2}^{2}, {\upsigma }_{\text{e}_1}^{2}$ and ${\upsigma }_{\text{e}_2}^{2}$ are the variances of additive, maternal permanent environmental and residual effects for traits 1 and 2, respectively;${\upsigma }_{\text{a}_1\text{a}_2}$, ${\upsigma }_{\text{m}_1\text{ m}_2}$ and ${\upsigma }_{\text{e}_1\text{e}_2}$ are the covariances of the additive, maternal permanent environmental and residual effects between traits 1 and 2, respectively. Significance of the genetic correlations was determined using an approximate 95% confidence interval, where the estimate was considered not significant if it fell within ± 2 times the standard errors from zero.

## Results

### Temporal alignment of fibre diameter and body weight data

**Fig. 2:**
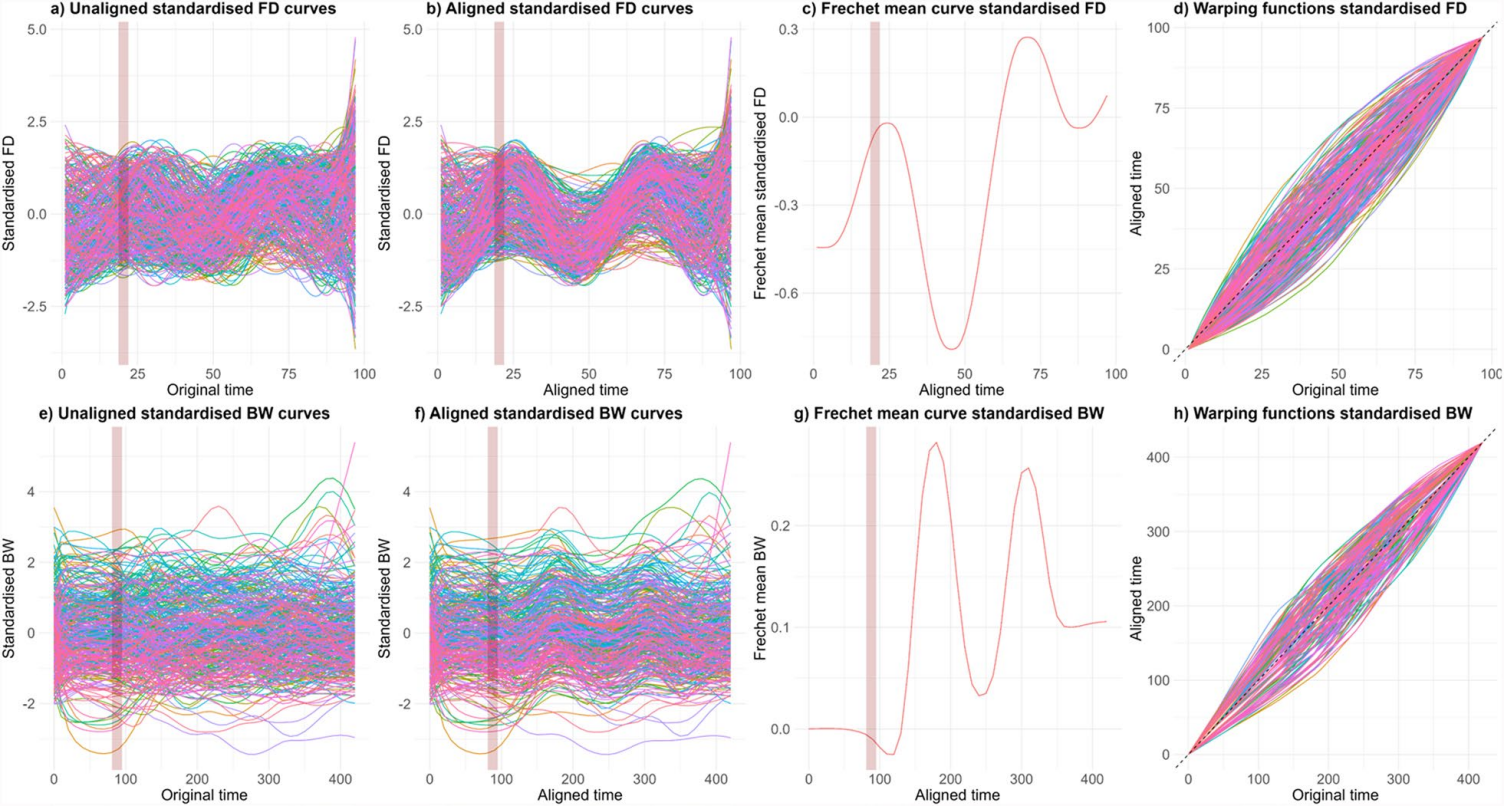
**a** Unaligned curves fibre diameter (FD), **b** aligned curves FD, **c** Frechet mean curve FD and **d** warping function of FD. The (**e**) unaligned curves body weight (BW), **f** aligned curves BW, **g** Frechet mean curves BW and (**f**) warping function BW from site year IN01_2008

The temporal alignment of longitudinal fibre diameter and body weight was conducted using the SRVF transformation, which removes the phase-based variation. The application of this method is novel to longitudinal traits in animal breeding, therefore, the effect of this method is demonstrated in Fig. 2 for the flock-year IN01_2008. Figure 2a and e show the standardised fibre diameter and body weight curves before the alignment process. Phase variation was observed because the animal curves show a similar overall shape, but the deviations occur at different time points. Figure 2b and f display the aligned fibre diameter and body weight functions using the SRVF transformation. Figure 2c and g show the Fréchet mean curve of the flock year to which the functions were aligned. The red shaded line indicates the average age at which weaning occurred in this flock year. The warping functions, which are the proportion of the phase-based variation that was removed from each of the fibre diameter and weight functions, are shown in Fig. 2d and h. The x-axis of the warping functions represents the original time points of the data, while the y-axis shows the corresponding aligned time points. The warping functions illustrate how the original time domain has been transformed to achieve the temporal alignment. Deviation from the identity line (y = x) indicates the degree of warping applied at each time point of the animal function. When the warping function of an animal's curve is above the identity line, the aligned time is greater than the original time for that segment of the curve (it has been stretched). In comparison, when the function is below the identity line, the aligned time is less than the original time for the segment, which means that the original function is mapped to an earlier time in the aligned function (compressed).

The degree of warping was adjusted using the lambda term. The optimal values of lambda chosen for standard fibre diameter and body weight were chosen to be 0.2 and 0.6, respectively, based on the genetic and phenotypic correlations between traits definitions presented in (see Additional file 1, Table S1). The lambda value of zero was considered too flexible, producing genetic correlations that were more variable between traits, ranging from 0.53 to 1.00. A lambda of 0.2 for fibre diameter showed a high genetic correlation with other values (0.4 and 0.6, ranging from 0.84 to 1.00), while a lambda of 0.6 for body weight showed a correlation ranging from 0.88 to 0.99. These chosen values were also graphically checked to ensure the alignment was biologically plausible.

### Comparison of the genetic parameters of potential resilience indicators

Summary statistics and genetic parameter estimates of resilience indicators are shown in Table 1. The heritability estimates of resilience indicators created in response to the known challenge of weaning (ROC_response, ROC_recovery and ABC) were low in both fibre diameter and body weight data, ranging between 0.04 and 0.08, except for BW_ABC, which showed a moderate heritability of 0.18 ± 0.04. The heritabilities for resilience indicators to unknown disturbance events were also low, ranging between 0.02 and 0.13 in both data series.

**Table 1:** Descriptive statistics and genetic parameters (additive variance (σ_a_^2^), maternal permanent environmental variance (σ_m_^2^), error variance (σ_e_^2^) and heritability (h^2^ ± SE)) of potential resilience indicator traits from fibre diameter (FD) and body weight (BW)

Trait	n	Mean (SD)	Range	σ_a_^2^	σ_m_^2^	σ_e_^2^	h^2^ ± SE
FD_Lnvar	6495	− 1.17 (0.81)	− 4.85–0.78	0.03	0.01	0.25	0.10 ± 0.02
FD_Auto	6495	0.87 (0.07)	0.44–0.98	0.00	NS^2^	0.00	0.03 ± 0.01
FD_Skewness	6495	0.08 (0.74)	− 2.78–3.35	0.04	0.03	0.45	0.07 ± 0.01
FD_ABS	6495	0.05 (0.02)	0.01–0.13	0.00	NS	0.00	0.05 ± 0.01
FD_ROC_response	4115	− 0.03 (0.04)	− 0.2–0.21	0.00	NS	0.00	0.06 ± 0.02
FD_ROC_recovery	4115	0.02 (0.04)	− 0.12–0.21	0.00	NS	0.00	0.08 ± 0.02
FD_ABC	4115	− 4.34 (13.02)	− 74.22–58.13	2.02	5.26	71.64	0.04 ± 0.02
BW_Lnvar	6222	− 2 (0.99)	− 6.80–1.10	0.05	0.09	0.78	0.06 ± 0.02
BW_Auto	6222	0.71 (0.16)	0.01–0.95	0.00	0.00	0.02	0.02 ± 0.02
BW_Skewness	6222	0.01 (1.00)	− 3.99–4.04	0.12	0.09	0.76	0.13 ± 0.02
BW_ABS	6222	0.08 (0.03)	0.01–0.38	0.00	NS	0.00	0.06 ± 0.02
BW_ROC_response	3826	0.00 (0.01)	− 0.08–0.03	0.00	NS	0.00	0.06 ± 0.02
BW_ROC_recovery	3826	0.00 (0.01)	− 0.03–0.06	0.00	NS	0.00	0.05 ± 0.02
BW_ABC	3826	− 2.58 (75.13)	− 339.82–321.86	1270	2820	3029	0.18 ± 0.04

^1^*Lnva*r  natural log variance of the deviation, *Auto*  lag1 Autocorrelation of the deviation, *Skewness*  skewness of the deviation, *ABS*  Absolute change in the deviation, *ROC_response*  rate of change during the response phase of the weaning challenge, *ROC_recovery*  rate of change during the recovery phase of the weaning challenge, *ABC*  area between curves during the weaning challenge. ^*2*^*NS*  non-significant

**Fig. 3:**
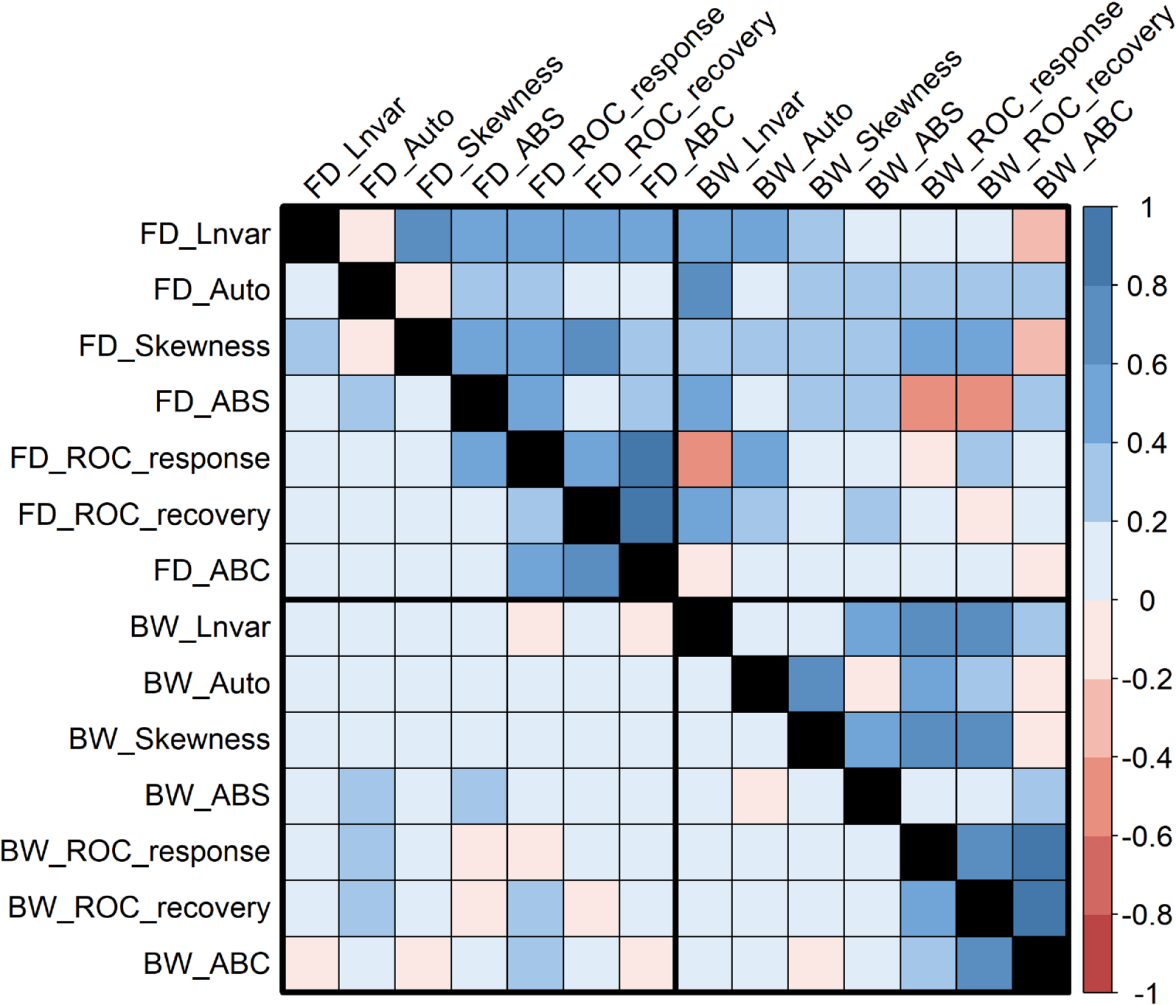
Genetic (above the diagonal) and phenotypic (below the diagonal) correlation between resilience indicators from longitudinal fibre diameter (FD) and body weight (BW). Standard errors of the genetic and phenotypic correlations ranged between 0.01 to 0.17 and 0.01 to 0.04, respectively. Abbreviations, Lnvar = natural log variance of the deviation, Auto = lag1 autocorrelation of the deviation, Skewness = skewness of the deviation, ABS = Absolute change in the deviation, ROC_response = rate of change during the response phase of the weaning challenge, ROC_recovery = rate of change during the recovery phase of the weaning challenge, ABC = area between curve during the weaning challenge

The genetic and phenotypic correlations between resilience indicator traits are presented in Fig. 3. The trait FD_Lnvar showed moderate to high positive correlation with seven other traits (FD_Skewness, FD_ABS, FD_ROC_response, FD_ROC_recovery, BW_Lnvar, BW_Auto and BW_Skewness in the range of 0.36 ± 0.02 to 0.76 ± 0.02. Within the fibre diameter resilience traits, the three traits that were used to describe how animals respond to the weaning disturbance (FD_ROC, response and recovery and FD_ABC) were most moderately correlated with the other indicator traits FD_Lnvar, FD_Skewness, FD_Auto and FD_ABS (range 0.19 ± 0.02 to 0.68 ± 0.02). Similar trends were shown within the body weight traits except for BW_ABS, which was only lowly correlated with the three weaning response traits (0.01 ± 0.02 to 0.21 ± 0.02). Regarding the correlation between fibre diameter and body weight resilience traits, most traits were low to moderately correlated, particularly between the traits (Lnvar, Auto, Skewness and ABS: 0.18 to 0.66). Lower genetic correlations were shown between FD_Auto and BW_Auto (0.01 ± 0.02) as well as FD_ABC with all other bodyweight resilience indicators (− 0.11 to 0.15). Phenotypic correlations between resilience traits were mostly low to negligible (Fig. 3). Full details are shown in (see Additional file 2, Table S2).

### Associations between resilience indicators, health and production traits

The descriptive statistics and heritabilities of the health and production traits are shown in Table 2. The production traits (CFW, FDCV, MFD, SS, SL, EMD, FAT and WT) had a similar number of trait records as the resilience phenotypes (~ 6000), whereas several of the health traits (DERM and BRWR) had fewer trait records (~ 3000). The heritability estimates of the health-related traits (DERM, DAG, BRWR, FLROT and CS) were low to moderate (0.10 ± 0.02 to 0.35 ± 0.02), while the heritabilities of the production (CFW, MFD, FDCV, SS, SL, EMD, FAT and WT) were higher (0.35 ± 0.03 to 0.68 ± 0.02).

**Table 2:** Descriptive statistics and heritabilities (h^2^ ± SE) of health and production traits

Trait	n	Mean (SD)	Range	h^2^ ± SE^a^
DERM	3025	0.83 (0.56)	1–5	0.25 ± 0.03
DAG	4330	1.82 (1.03)	1–5	0.15 ± 0.02
BRWR	2788	2.59 (0.89)	1–5	0.35 ± 0.02
FLROT	5620	1.71 (1.22)	1–5	0.10 ± 0.02
CS	4387	2.86 (0.43)	1–4.5	0.21 ± 0.02
WEC	4985	9.15 (4.75)	0–38.00	0.20 ± 0.02
CFW	6278	2.40 (0.73)	0.54–5.57	0.52 ± 0.02
MFD	6758	16.79 (1.79)	12.50–33.7	0.68 ± 0.02
FDCV	6734	18.28 (2.71)	11.70–31.30	0.47 ± 0.02
SS	6025	33.45 (12.15)	2–88	0.42 ± 0.02
SL	6029	87.23 (16.72)	23–160	0.48 ± 0.02
EMD	5042	21.82 (4.05)	10–34	0.35 ± 0.03
FAT	5042	2.54 (0.89)	0.5–9.0	0.44 ± 0.02
WT	5042	37.65 (7.36)	16.5–71.2	0.45 ± 0.02

*n*  number of records, *DERM*  wool dermatophilosis, *DAG*  dag score, *BRBW*  breech wrinkle, *FLROT*  fleece rot score, *CS*  body condition score, *WEC*  cube root worm egg count, *CFW*  clean fleece weight, *MFD*  mean fibre diameter, *FDCV*  fibre diameter coefficient of variation, *SS*  staple strength, *SL*  staple length, *EMD*  eye muscle depth, *FAT*  Fat depth, *WT*  Body weight

^a^Heritabilities estimates based on the mean of the heritabilities obtained from the bivariate analysis

Table 3 provides the genetic correlations between resilience indicators and health-related traits. The correlation between CS and FD_Lnvar was negative (− 0.23 ± 0.08), while weak positive correlations were observed between CS and FD_Auto, FD_ROC_recovery, BW_Skewness, BW_ABS, and BW_ABC, ranging from 0.10 ± 0.02 to 0.27 ± 0.11. The trait DERM was low but significantly positively correlated with several traits, including FD_Lnvar, FD_Auto, FD_ABS, FD_ROC_recovery, BW_Auto and BW_ABC in the range of 0.06 ± 0.02 to 0.09 ± 0.01. Indicators related to flystrike, DAG and BRWR were not significantly correlated with any resilience indicator, except for BRWR and FD_Auto (0.12 ± 0.02). The indicator of internal parasites, WEC, was also not correlated to resilience indications except for FD_Skewness (− 0.36 ± 0.13). The remaining genetic correlations between resilience indicators and health traits were low and not significantly different from zero. The phenotypic correlations between resilience indicators and health traits were low (− 0.06 ± 0.01 to 0.04 ± 0.01) and are presented in (see Additional file 3, Table S3).

**Table 3:** Genetic correlations (± SE) between resilience indicator traits and health traits in sheep

Resilience trait	Health traits					
	DERM	DAG	BRWR	FLROT	CS	WEC
FD_Lnvar	0.09 ± 0.01	0.08 ± 0.03	0.25 ± 0.16	0.30 ± 0.10	− 0.23 ± 0.08	− 0.14 ± 0.12
FD_Auto	0.08 ± 0.02	0.00 ± 0.01	0.12 ± 0.02	0.05 ± 0.01	0.25 ± 0.03	− 0.07 ± 0.01
FD_Skewness	0.02 ± 0.02	0.02 ± 0.02	0.08 ± 0.02	0.29 ± 0.12	− 0.20 ± 0.14	− 0.36 ± 0.13
FD_ABS	0.07 ± 0.01	0.07 ± 0.01	− 0.02 ± 0.01	0.03 ± 0.02	− 0.03 ± 0.02	− 0.15 ± 0.10
FD_ROC_response	0.09 ± 0.01	− 0.09 ± 0.03	− 0.00 ± 0.04	− 0.09 ± 0.03	− 0.02 ± 0.04	− 0.05 ± 0.02
FD_ROC_recovery	0.06 ± 0.04	− 0.03 ± 0.01	0.04 ± 0.01	0.07 ± 0.02	0.10 ± 0.02	0.03 ± 0.02
FD_ABC	0.01 ± 0.01	− 0.02 ± 0.02	− 0.05 ± 0.05	− 0.01 ± 0.02	− 0.13 ± 0.06	− 0.11 ± 0.12
BW_Lnvar	0.01 ± 0.02	0.04 ± 0.02	0.06 ± 0.03	0.14 ± 0.13	0.21 ± 0.15	− 0.14 ± 0.13
BW_Auto	0.07 ± 0.02	0.01 ± 0.03	− 0.03 ± 0.03	0.21 ± 0.12	0.04 ± 0.01	0.06 ± 0.02
BW_Skewness	0.03 ± 0.05	0.05 ± 0.03	0.05 ± 0.02	0.24 ± 0.10	0.27 ± 0.11	− 0.10 ± 0.03
BW_ABS	0.04 ± 0.02	− 0.03 ± 0.01	− 0.03 ± 0.01	0.04 ± 0.01	0.20 ± 0.03	0.02 ± 0.01
BW_ROC_response	0.03 ± 0.01	0.01 ± 0.02	− 0.01 ± 0.02	0.01 ± 0.01	0.02 ± 0.01	0.00 ± 0.04
BW_ROC_recovery	0.03 ± 0.01	0.02 ± 0.02	0.07 ± 0.02	− 0.01 ± 0.01	0.00 ± 0.01	0.00 ± 0.01
BW_ABC	0.06 ± 0.02	0.01 ± 0.02	0.05 ± 0.02	0.01 ± 0.02	0.21 ± 0.04	0.09 ± 0.04

*FD*  fibre diameter, *BW*  body weight, *Lnvar*  natural log variance of the deviation, *Auto*  lag1 Autocorrelation of the deviation, *Skewness*  skewness of the deviation, *ABS*  Absolute change in the deviation, *ROC_response*  rate of change during the response phase of the weaning challenge, *ROC_recovery*  rate of change during the recovery phase of the weaning challenge, *ABC*  area between curve during the weaning challenge, *DERM*  wool dermophilisis, *DAG*  dag score, *BRWR*  breech wrinkle, *FLROT*  fleece rot score, *CS*  body condition score, *WEC*  Worm egg count

Table 4 presents the genetic correlations between resilience indicators and production traits. Overall, resilience traits showed weak to moderate correlations with production traits. Among the fibre diameter resilience traits, FD_Lnvar and FD_Skewness exhibited moderate negative correlations with MFD and SS (− 0.51 to − 0.30) and positive correlations with FDCV and SL (0.15 to 0.55). Both FD_Auto and FD_ABS showed generally low correlations with production traits, with the strongest positive correlation observed between FD_ABS and CFW (0.17 ± 0.02) and the strongest negative correlation between FD_ROC_response and FAT (− 0.33 ± 0.10). The three traits related to weaning response (FD_ROC_response, FD_ROC_recovery and FD_ABC) had low correlations with production traits and were associated with higher standard errors. Body weight resilience indicators showed weak to moderate positive correlations with fleece traits (CFW, MFD, FDCV, SS and SL), with the strongest observed between BW_Skewness and FDCV (0.35 ± 0.10). More consistent correlations were observed across body weight resilience indicators and carcass traits (EMD, FAT and WT), estimates ranging from − 0.02 to − 0.38. The phenotypic correlation matrix between resilience indicators and production traits is provided in (Additional file 4, Table S4).

**Table 4:** Genetic correlations (± SE) between resilience indicator traits and production traits

Resilience trait	Production traits							
	CFW	MFD	FDCV	SS	SL	EMD	FAT	WT
FD_Lnvar	− 0.09 ± 0.02	− 0.32 ± 0.08	0.15 ± 0.09	− 0.51 ± 0.09	0.55 ± 0.09	− 0.16 ± 0.02	− 0.19 ± 0.11	0.08 ± 0.02
FD_Auto	0.12 ± 0.02	0.09 ± 0.02	0.03 ± 0.01	0.01 ± 0.02	0.01 ± 0.02	− 0.02 ± 0.02	− 0.31 ± 0.07	0.13 ± 0.03
FD_Skewness	− 0.10 ± 0.02	− 0.44 ± 0.09	0.44 ± 0.10	− 0.30 ± 0.10	0.55 ± 0.10	− 0.15 ± 0.04	− 0.13 ± 0.10	− 0.05 ± 0.02
FD_ABS	0.17 ± 0.02	0.10 ± 0.02	− 0.06 ± 0.03	− 0.02 ± 0.03	− 0.04 ± 0.02	− 0.06 ± 0.02	− 0.13 ± 0.02	− 0.07 ± 0.02
FD_ROC_response	0.41 ± 0.12	− 0.21 ± 0.12	− 0.08 ± 0.02	0.01 ± 0.02	− 0.09 ± 0.02	− 0.03 ± 0.02	− 0.33 ± 0.10	− 0.14 ± 0.02
FD_ROC_recovery	0.44 ± 0.14	0.13 ± 0.04	− 0.09 ± 0.03	− 0.03 ± 0.02	− 0.11 ± 0.04	− 0.04 ± 0.02	− 0.34 ± 0.09	0.03 ± 0.02
FD_ABC	0.13 ± 0.15	0.10 ± 0.12	0.03 ± 0.15	− 0.07 ± 0.10	0.10 ± 0.12	− 0.15 ± 0.02	− 0.10 ± 0.03	− 0.05 ± 0.04
BW_Lnvar	0.22 ± 0.10	0.05 ± 0.08	0.26 ± 0.10	− 0.01 ± 0.01	− 0.02 ± 0.01	− 0.14 ± 0.13	− 0.16 ± 0.14	− 0.33 ± 0.06
BW_Auto	0.15 ± 0.03	0.09 ± 0.06	0.17 ± 0.09	− 0.03 ± 0.02	− 0.01 ± 0.02	− 0.04 ± 0.01	− 0.23 ± 0.07	− 0.09 ± 0.04
BW_Skewness	− 0.20 ± 0.08	− 0.37 ± 0.07	0.35 ± 0.09	− 0.05 ± 0.01	− 0.02 ± 0.02	− 0.05 ± 0.02	− 0.13 ± 0.07	− 0.37 ± 0.05
BW_ABS	0.30 ± 0.04	0.13 ± 0.04	− 0.06 ± 0.01	− 0.05 ± 0.02	0.02 ± 0.02	− 0.02 ± 0.04	− 0.21 ± 0.02	− 0.38 ± 0.07
BW_ROC_response	0.13 ± 0.03	− 0.05 ± 0.02	− 0.02 ± 0.03	− 0.09 ± 0.04	0.03 ± 0.02	− 0.03 ± 0.02	− 0.13 ± 0.04	0.04 ± 0.03
BW_ROC_recovery	− 0.06 ± 0.02	0.06 ± 0.02	− 0.03 ± 0.02	− 0.05 ± 0.02	0.04 ± 0.02	− 0.02 ± 0.02	0.06 ± 0.02	0.02 ± 0.02
BW_ABC	0.15 ± 0.05	− 0.04 ± 0.02	0.10 ± 0.05	− 0.09 ± 0.06	0.14 ± 0.04	− 0.12 ± 0.03	− 0.22 ± 0.04	0.82 ± 0.04

*FD*  fibre diameter, *BW*  body weight, *Lnvar*  natural log variance of the deviation, *Auto*  lag1 Autocorrelation of the deviation, *Skewness*  skewness of the deviation, *ABS*  Absolute change in the deviation, *ROC_response*  rate of change during the response phase of the weaning challenge, *ROC_recovery*  rate of change during the recovery phase of the weaning challenge, *ABC*  area between curve during the weaning challenge, *CFW*  clean fleece weight, *MFD*  mean fibre diameter, *FDCV*  fibre diameter coefficient of variation, *SS*  staple strength, *SL*  staple length, *EMD*  eye muscle depth, *FAT*  Fat depth, *WT*  Body weight

## Discussion

Increasing resilience has become a key objective in modern sheep breeding, yet there remains a lack of effective methods for quantifying resilience. This study focused on assessing resilience traits derived from deviations in wool fibre diameter and body weight within longitudinal data. The resilience traits were found to be low to moderately heritable. Notably, traits such as FD_Lnvar and BW_Lnvar showed strong correlations with traits describing the responses to weaning, indicating their potential as indicators of resilience. Additionally, the correlations between resilience traits and both health and production traits were low to moderately negative, suggesting that less resilient animals might face more health issues and exhibit lower production levels.

### Adjusting for phase variation

In this study, the SRVF transformation was applied to reduce phase variability before calculating resilience phenotypes. This approach aimed to improve the interpretation of amplitude features, which was important for developing resilience traits in relation to a perceived disturbance, in this case, weaning. Previous studies have highlighted challenges in interpreting resilience phenotypes from fibre diameter variation, partly due to phase misalignment [23].

Within the SRVF method, the choice of lambda significantly impacts alignment outcomes and represents a potential limitation. Higher lambda values produce smoother, more rigid warping functions that reduce overfitting but may fail to correct phase variability. Conversely, lower values allow greater flexibility but risk overfitting to noise. Selecting an appropriate lambda remains largely unexplored in real-world applications, yet it is essential for meaningful alignments. In this study, genetic correlations remained stable with moderate penalties (lambda of 0.2, 0.4, 0.6) but decreased under fully flexible alignment (lambda = 0). Some traits, such as ABC, appear more sensitive to lambda choice as greater curve flexibility increased its value, while Lnvar, capturing overall variability, remained less affected.

Alignment was visually more consistent for fibre diameter than body weight functions, as shown in the warping function and comparisons between aligned and unaligned functions (Fig. 2). This discrepancy likely stems from several factors: body weight data was collected from growing animals, leading to greater mean differences despite standardisation. Additionally, body weight deviations were smaller and less synchronised within flock-year groups. Given these factors, a higher lambda (0.6) was used for body weight to prevent unrealistic alignment, whereas a lower lambda (0.2) was used for fibre diameter. These values were validated against biological expectations; plus or minus 25 mm for fibre diameter (based on wool growth rates and measurement error [46]) and a threshold of 54 days for body weight (reflecting the maximum age difference between individuals). Collectively, using the SRVF on body weight data may be unnecessary, though it provided a useful comparison with fibre diameter. The technique may be more suited to capturing resilience in adult sheep, where it isn’t confounded with growth and where deviation in weight primarily relates to condition loss or gain. Future body weight analyses could also use simpler alternatives like mechanistic modelling (e.g., Gompertz functions).

A final consideration is that phase variations may contain valuable information about an animal’s response to disturbances, which could be lost during transformation. Differences in reaction speed and timing may be biologically meaningful and warrant further investigation. However, in this study, the impact on resilience indicators appeared minimal, as trait correlations remained consistent across different lambda values.

### Genetic parameters of resilience indicators

The heritability estimates of resilience indicators developed from longitudinal wool fibre diameter and body weight were low, ranging between 0.03 to 0.18. The heritabilities were similar between the traits describing overall variability in the curves and response to weaning. The estimates of Lnvar, Auto and Skewness were similar to the estimates of Gorssen et al. [9] in pigs and Berghof et al. [12] in chickens. Higher heritability estimates of the same trait definition have been provided by Poppe et al. [4] and Rodrigues et al. [47] for milk yield and growth in dairy and beef cattle, respectively (heritabilities of approximately 0.20). The difference in heritabilities between studies could be attributed to differences in sampling interval or the responsiveness of the longitudinal trait to disturbances. Together, the heritabilities and variance components in the current study suggest that the resilience indicators have some suitable genetic variation that could be used to potentially improve the resilience of sheep through selection.

### Resilience indicators based on the response to a known disturbance compared to overall variability

Few studies have explored whether resilience indicators, such as Lnvar, Auto, or Skewness, are genuinely related to animal responses to known disturbances. While previous research often assumes that deviations in trait performance over time reflect a lack of resilience to short-term stress events, this assumption remains unsubstantiated in most datasets. This study investigated the genetic correlation between resilience indicators describing trait fluctuations over the entire wool growth period (representing unknown disturbances) and resilience to the specific disturbance of weaning (measured by FD_ROC_response and recovery and ABC in fibre diameter data). The findings revealed mostly moderate to strong positive correlations between these indicators. For instance, a strong positive correlation was observed between FD_Lnvar and FD_ROC_response. Resilient sheep tended to exhibit low variance and low rates of change in response, maintaining stable responses (Lnvar around zero). In contrast, sensitive sheep showed higher variance and rates of change, indicating less stability and potentially higher reactivity to disturbances. These results suggest that animals exhibiting greater rates of change in response and recovery to weaning also respond similarly to unknown disturbances. This conclusion aligns with the findings of Poppe et al. [48], who reported high to moderate genetic correlations between milk yield Lnvar and Auto with traits related to responses to a known heat wave event in dairy cattle.

Within the body weight resilience indicators, higher genetic correlations were observed between BW_Lnvar, BW_Auto and BW_Skewness with BW_ROC_response and recovery. The remaining trait pairs between BW_ABS showed lower genetic correlations. It is possible that for this trait, the weaning period does not have a substantial effect on body weight, particularly considering that the animals are managed in a research environment where growth targets are managed. This is in contrast to other species, such as pigs, where weaning at younger ages (3–4 weeks) has been shown to elicit a greater physiological challenge, which could also be useful for examining resilience [10].

### Resilience indicators from fibre diameter or body weight

Many recent studies on resilience have used a single data series to characterise animals' ability to minimise the effects of environmental disturbances, often focusing on just one biological parameter. However, it has been suggested that utilising multiple data sources could enhance understanding of resilience, given the complex dynamics of trait responses. In this study, the genetic correlations between fibre diameter and body weight resilience indicators varied in strength and direction. FD_Lnvar, FD_Skewness, and FD_Auto showed moderate positive correlations with most BW resilience traits (0.30–0.42), suggesting some consistency in resilience patterns across traits. In contrast, FD_ABS showed a larger negative correlation with BW_ROC_response (− 0.53) and BW_ROC_recovery (− 0.46), indicating potential differences in how phase variation is captured in fibre diameter versus body weight. Other fibre diameter and body weight trait pairs displayed low correlations. These findings may be influenced by several factors such as the trait's responsiveness to disturbances, measurement differences, response timescales and resource allocation across different body functions.

In the current dataset, deviations in body weight appear less prominent and less responsive to disturbances compared to fibre diameter. Fibre diameter responds more quickly to disturbances, while body weight changes tend to occur gradually, reflecting long-term adaptation with less immediate variability. For example, Schlink and Dollin [49] demonstrated that fibre diameter can vary by as much as 8 µm over two days of wool growth, whereas similar changes are not observed in body weight. The differences in this study may be partly due to the physiological trade-offs between body functions. According to Freer et al. [15], there is a hierarchical partitioning of energy and protein between wool, meat and fat, with resources first allocated to maintenance, followed by wool production, and then muscle and fat deposition. This means that fibre diameter is more sensitive to energy and protein imbalances compared to weight changes. During a challenge, the animals are likely to repartition energy from fibre production while trying to conserve body weight. Additionally, the scale of recording differs between the two data types; body weight was estimated at 10-day intervals, while fibre diameter was estimated at 1 mm, equivalent to about three days of wool growth. This might cause the short-term variation in body weight to average out. These factors may contribute to the lower correlation between certain fibre diameter and body weight resilience traits, while also acknowledging that some of the resilience indicators were moderately correlated. Abdelkrim et al. [8] observed similar results when comparing deviations in milk yield and body weight.

### Association between resilience indicators and health indicator traits

The objective of calculating the correlation between resilience indicators and health traits was to assess the biological significance of deviations captured by resilience phenotypes to determine if they accurately reflect resilience. This validation is challenging due to the lack of a direct measure or gold standard for measuring resilience, although other studies suggest that resilience can be related to fewer health and disease incidences [6]. With all other factors being equal, more resilient sheep are expected to have fewer health problems and have greater reproduction rates and longevity.

Several of the resilience indicator traits developed in this study showed moderate genetic correlation with CS, suggesting that animals with greater variation in fibre diameter and body weight tend to have lower CS after the weaning challenge. Poppe et al. [4] reported similar genetic correlations between body condition scores in dairy cattle with milk yield Lnvar, Skewness and Auto (range − 0.06 to − 0.41), supporting the idea that resilience is associated with resource allocation and trade-offs between body functions. Despite the correlation between resilience indicators and CS in the current study, questions remain around the suitability of measuring condition scores on young animals. The majority of current knowledge about condition score in sheep comes from adult ewes, and it is unclear if body condition score is a reliable indicator of health and resource accumulation in young animals. It is noted that the correlation with condition score is similarly reflected in the correlations with EMD and FAT, which are the quantitative measures of muscle and fat deposition. Further studies could verify these correlations by calculating the resilience phenotypes in older animals.

It is also acknowledged here that quite a few of the correlations between resilience and DAG, BRWR, FLROT and DERM scores were weak or were accompanied by larger standard errors. These correlations could be affected by the low number of trait records and limited expression of the disease traits under the research management protocols. Validation of these correlations in a larger population of animals managed under commercial production conditions is required, where challenges are observed and recorded. While these correlations are low, in Merino sheep, they may still have important practical considerations as Merino yearlings are notoriously prone to ill health, which can lead to high mortalities (> 10% under commercial conditions) and is known to have a genetic component [50, 51]. While the relationship between yearling mortality and resilience traits was not examined here, further studies could determine whether low-resilience families have a higher number of yearling mortalities. This would provide further validation of these resilience indicators against accumulated consequences of resilience (e.g., survival and longevity) as advocated by Friggens et al. [6].

### Genetic correlations between resilience indicators and production traits

The genetic correlation between resilience indicators and eight economically important production traits included in Australia’s national genetic evaluation was calculated to assess whether higher resilience is associated with productivity. Moderate favourable correlations were observed between FD_Lnvar, BW_Lnvar, FD_Skewness and BW_Skewness with FDCV, suggesting that more resilient animals tend to have lower overall FDCV. Similarly, FD_Lnvar and FD_Skewness were favourably correlated with SS, an important wool quality characteristic. Preston and Hatcher [52] also reported similar relationships between the along-fibre coefficient of variation, overall FDCV, and SS. This result is expected, as variation in fibre diameter along a staple directly contributes to both FDCV and fibre strength.

Additionally, several studies have suggested that an animal’s MFD influences its wool growth responsiveness to environmental changes. Sheep with lower MFD reportedly experience less fluctuation in fibre diameter in response to nutritional changes compared to those with higher MFD [21]. This is likely due to a scale effect, where the same disturbance leads to a proportionally larger decline in fibre diameter in high MFD animals, even if the relative impact on fibre diameter is similar across different MFD groups. However, this pattern was not observed in the current study, aligning with findings by Safari et al. [53] and Huisman and Brown [54], who reported low negative genetic correlations between MFD and overall FDCV of approximately -0.10 and − 0.17, respectively.

Other notable correlations include the weak but favourable relationship between body weight and fibre diameter resilience indicators (e.g., Skewness or ABS) with EMD, FAT and WT. This suggests that more resilient individuals, with less variation in body weight or fibre diameter, are likely to be heavier and have greater EMD and fat deposition. A potential explanation for the negative genetic correlation is an energy allocation trade-off. Animals with more body fat may have better energy reserves and more stable metabolic regulation, allowing them to cope more effectively with stressors such as nutritional fluctuations, disease and environmental challenges. Alternatively, because selection has favoured higher fat, muscle and weight, the population may have adapted in a way that links greater fat deposition with more stable performance. Similarly to the current study, Ferguson et al. [55] found that ewe liveweight profiles were consistently associated with changes in wool fibre diameter variability and reproductive outcomes. However, the causal direction of the relationship between resilience and production is not clear. In complex traits like resilience, multiple biological pathways interact, making it difficult to distinguish cause from effect. Graphical models, such as structural equation models and Bayesian networks, provide a framework for exploring these relationships by testing, for instance, whether fat directly influences resilience or whether resilience affects fat accumulation, rather than simply detecting a correlation. This could be an area that is worth further exploration.

Collectively, the correlation between resilience indicators and production traits was favourable, meaning that it would be possible to include resilience phenotypes in the breeding objective without having an antagonistic relationship with the key production traits. A similar result was also shown in other species, including pigs, chickens and dairy cattle [3, 4, 8]. Only one resilience trait, BW_ABC, showed a high genetic correlation with body weight, suggesting it reflects the animal's overall body weight rather than resilience. Similar challenges have been encountered in studies of milk yield, where variance in yield is often scaled according to overall milk production. In such cases, partial genetic correlations have been employed to explore the relationship between resilience phenotypes and other traits [4]. While a similar approach could be applied in this study, it does not appear necessary, given that all other resilience indicators show low correlations with the mean of the trait (e.g., MFD or WT). It is also worth noting that the heritabilities of the production traits observed in this study were slightly higher than those reported by other authors [53], particularly for traits related to wool production and quality (CFW, FDCV, MFD, SS and SL), which may have influenced the results. A re-examination of these correlations in a larger, more genetically diverse population of sheep may be necessary.

## Conclusions

This study demonstrated the ability to account for temporal variation in longitudinal data before developing potential resilience indicators to describe both general resilience and specific resilience to the weaning disturbance, using two data sources: fibre diameter and body weight. The resilience indicators were found to be heritable, indicating they could be used for selection in breeding programs. For fibre diameter data, resilience indicators related to unknown disturbances were similar to those describing response and recovery from the specific disturbance of weaning. The genetic correlations between resilience indicators derived from fibre diameter and body weight were moderate for most traits, suggesting that animals may utilise diverse pathways to overcome disturbances. The correlation between resilience traits and health traits was mostly zero, except for body condition score. Resilience traits were favourably correlated to several production traits, including traits related to body reserves (EMD, FAT and WT) and wool quality. Fibre diameter appears to be the preferred data series, with Lnvar likely being the preferred trait, based on heritabilities, genetic correlations between different resilience indicators, and correlations with health and production traits. Practically, longitudinal fibre diameter records can also be attained relatively easily due to their cost and ease of collection. Historically, the collection of any longitudinal production records in extensive systems has been a barrier to developing resilience indicators for these animals.

## Supplementary Information

Additional file 1: Table S1. Genetic and phenotypic correlations between resilience traits estimated with four lambda values (0, 0.2, 0.4 and 0.6) used to adjust the warping function during the temporal alignment using the Square Root Velocity Function transformation.

Additional file 2: Table S2. Genetic (above the diagonal) and phenotypic correlations (below the diagonal) between resilience indicators derived from standardised wool fibre diameter and body weight in sheep.

Additional file 3: Table S3. Phenotypic correlations (±SE) between resilience indicators derived from standardised wool fibre diameter and body weight and health traits in sheep.

Additional file 4: Table S4. Phenotypic correlations (±SE) between resilience indicators derived from standardised wool fibre diameter and body weight and production traits in sheep.

## Data Availability

The data used in this study have not been deposited in an official repository. The data are owned by Meat and Livestock Australia. Access to the data can be negotiated upon request.
