# Supplementary material for: Estimating the genetic parameters of resilience toward known and unknown disturbances in sheep using wool fibre diameter and body weight variability
Source: Genet Sel Evol. 2025 Jul 14;57:38. doi: 10.1186/s12711-025-00983-1 (PMC12257680; doi:10.1186/s12711-025-00983-1)
Supplement: Supplementary file 1 — Additional file 1: Table S1. Genetic and phenotypic correlations between resilience traits estimated with four lambda values (0, 0.2, 0.4 and 0.6) used to adjust the warping function during the temporal alignment using the Square Root Velocity Function transformation. [file 12711_2025_983_MOESM1_ESM.docx]

Additional file 1, Table S1

Genetic and phenotypic correlations between resilience traits estimated with four lambda values (0, 0.2, 0.4 and 0.6) used to adjust the warping function during the temporal alignment using the Square Root Velocity Function transformation.

| **Standardised wool fibre diameter** | | | | | | |
| --- | --- | --- | --- | --- | --- | --- |
| **Genetic correlations** | | | | | | |
| **Trait** | **lambda0_0.2** | **lambda0_0.4** | **lambda0_0.6** | **lambda0.2_0.4** | **lambda0.2_0.6** | **lambda0.4_0.6** |
| **Lnvar** | 0.95 ±0.09 | 0.94 ±0.11 | 0.99 ±0.13 | 0.99 ±0.01 | 0.92 ±0.01 | 0.99 ±0.00 |
| **Auto** | 0.54 ±0.01 | 0.86 ±0.01 | 0.84 ±0.01 | 0.92 ±0.01 | 0.96 ±0.01 | 0.96 ±0.01 |
| **Skewness** | 0.99 ±0.02 | 1.00 ±0.07 | 0.96 ±0.08 | 1.00 ±0.01 | 0.98 ±0.01 | 1.00 ±0.01 |
| **Abs** | 0.96 ±0.00 | 0.96 ±0.00 | 0.95 ±0.00 | 0.96 ±0.00 | 0.96 ±0.00 | 0.96 ±0.00 |
| **ROC_resp** | 0.89 ±0.01 | 0.89 ±0.01 | 0.53 ±0.03 | 0.96 ±0.00 | 0.95 ±0.00 | 0.96 ±0.00 |
| **ROC_reco** | 0.90 ±0.00 | 0.90 ±0.00 | 0.79 ±0.00 | 0.95 ±0.00 | 0.92 ±0.00 | 0.95 ±0.00 |
| **ABC** | 0.53 ±0.32 | 0.58 ±0.32 | 0.54 ±0.27 | 0.96 ±0.10 | 0.84 ±0.14 | 0.87 ±0.12 |
| **Phenotypic correlation** | | | | | | |
| **Trait** | **lambda0_0.2** | **lambda0_0.4** | **lambda0_0.6** | **lambda0.2_0.4** | **lambda0.2_0.6** | **lambda0.4_0.6** |
| **Lnvar** | 0.07 ±0.02 | 0.07 ±0.02 | 0.07 ±0.02 | 0.14 ±0.01 | 0.10 ±0.03 | 0.17 ±0.01 |
| **Auto** | 0.17 ±0.01 | 0.55 ±0.02 | 0.53 ±0.02 | 0.69 ±0.01 | 0.69 ±0.02 | 0.69 ±0.01 |
| **Skewness** | 0.08 ±0.01 | 0.09 ±0.01 | 0.08 ±0.00 | 0.07 ±0.00 | 0.08 ±0.00 | 0.09 ±0.00 |
| **Abs** | 0.72 ±0.01 | 0.72 ±0.01 | 0.72 ±0.01 | 0.72 ±0.01 | 0.72 ±0.01 | 0.72 ±0.01 |
| **ROC_resp** | 0.71 ±0.01 | 0.72 ±0.01 | 0.38 ±0.01 | 0.71 ±0.01 | 0.71 ±0.01 | 0.71 ±0.01 |
| **ROC_reco** | 0.73 ±0.01 | 0.73 ±0.01 | 0.58 ±0.01 | 0.71 ±0.01 | 0.71 ±0.01 | 0.76 ±0.01 |
| **ABC** | 0.02 ±0.01 | 0.02 ±0.01 | 0.04 ±0.01 | 0.05 ±0.01 | 0.03 ±0.01 | 0.04 ±0.01 |
|  |  |  | **Body weight** |  |  |  |
| **Genetic correlations** | | | | | | |
| **Trait** | **lambda0_0.2** | **lambda0_0.4** | **lambda0_0.6** | **lambda0.2_0.4** | **lambda0.2_0.6** | **lambda0.4_0.6** |
| **Lnvar** | 0.95 ±0.06 | 0.96 ±0.04 | 0.96 ±0.04 | 1.00 ±0.01 | 0.99 ±0.01 | 0.88 ±0.01 |
| **Auto** | 0.92 ±0.01 | 0.92 ±0.01 | 0.91 ±0.01 | 0.95 ±0.01 | 0.95 ±0.01 | 0.96 ±0.01 |
| **Skewness** | 0.95 ±0.01 | 0.97 ±0.03 | 0.97 ±0.03 | 1.00 ±0.01 | 0.99 ±0.01 | 0.99 ±0.01 |
| **Abs** | 0.96 ±0.01 | 0.96 ±0.01 | 0.96 ±0.01 | 0.96 ±0.01 | 0.96 ±0.01 | 0.96 ±0.01 |
| **ROC_resp** | 0.95 ±0.01 | 0.96 ±0.01 | 0.96 ±0.01 | 0.96 ±0.01 | 0.96 ±0.01 | 0.95 ±0.01 |
| **ROC_reco** | 0.95 ±0.01 | 0.96 ±0.01 | 0.96 ±0.01 | 0.96 ±0.01 | 0.96 ±0.01 | 0.95 ±0.01 |
| **ABC** | 0.72 ±0.05 | 0.70 ±0.05 | 0.70 ±0.05 | 0.99 ±0.01 | 0.98 ±0.01 | 0.97 ±0.01 |
| **Phenotypic correlations** | | | | | | |
| **Trait** | **lambda0_0.2** | **lambda0_0.4** | **lambda0_0.6** | **lambda0.2_0.4** | **lambda0.2_0.6** | **lambda0.4_0.6** |
| **Lnvar** | 0.07 ±0.02 | 0.07 ±0.02 | 0.07 ±0.02 | 0.04 ±0.02 | 0.06 ±0.02 | 0.06 ±0.02 |
| **Auto** | 0.44 ±0.02 | 0.42 ±0.02 | 0.42 ±0.02 | 0.61 ±0.02 | 0.61 ±0.02 | 0.54 ±0.02 |
| **Skewness** | 0.14 ±0.02 | 0.14 ±0.02 | 0.15 ±0.02 | 0.04 ±0.02 | 0.05 ±0.02 | 0.18 ±0.02 |
| **Abs** | 0.69 ±0.00 | 0.69 ±0.00 | 0.69 ±0.00 | 0.69 ±0.00 | 0.69 ±0.00 | 0.69 ±0.00 |
| **ROC_resp** | 0.69 ±0.00 | 0.69 ±0.00 | 0.69 ±0.00 | 0.69 ±0.00 | 0.69 ±0.00 | 0.69 ±0.00 |
| **ROC_reco** | 0.69 ±0.00 | 0.69 ±0.00 | 0.69 ±0.00 | 0.69 ±0.00 | 0.69 ±0.00 | 0.69 ±0.00 |
| **ABC** | 0.22 ±0.03 | 0.23 ±0.03 | 0.21 ±0.03 | 0.38 ±0.02 | 0.34 ±0.03 | 0.37 ±0.03 |

Abbreviations, FD=fibre diameter, BW=body weight, Lnvar=natural log variance of the deviation, Auto=lag1 autocorrelation of the deviation, Skewness= skewness of the deviation, ABS=absolute change in the deviation, ROC_resp=rate of change during the response phase of the weaning challenge, ROC_reco= rate of change during the recovery phase of the weaning challenge, ABC=area between curve during the weaning challenge.
