# Supplementary material for: Estimating the genetic parameters of resilience toward known and unknown disturbances in sheep using wool fibre diameter and body weight variability
Source: Genet Sel Evol. 2025 Jul 14;57:38. doi: 10.1186/s12711-025-00983-1 (PMC12257680; doi:10.1186/s12711-025-00983-1)
Supplement: Supplementary file 2 — Additional file 2: Table S2. Genetic (above the diagonal) and phenotypic correlations (below the diagonal) between resilience indicators derived from standardised wool fibre diameter and body weight in sheep. [file 12711_2025_983_MOESM2_ESM.docx]

Additional file 2, Table S2

Genetic (above the diagonal) and phenotypic correlation (below the diagonal) between resilience indicators derived from standardised fibre diameter and body weight in sheep.

|  | **Fibre diameter** | | | | | | | **Body weight** | | | | | | |
| --- | --- | --- | --- | --- | --- | --- | --- | --- | --- | --- | --- | --- | --- | --- |
|  | **Lnvar** | **Auto** | **Skew** | **ABS** | **ROC_res** | **ROC_rec** | **ABC** | **Lnvar** | **Auto** | **Skewness** | **ABS** | **ROC_res** | **ROC_rec** | **ABC** |
| **FD_Lnvar** | 1 | -0.20±0.02 | 0.76±0.01 | 0.53±0.01 | 0.51±0.01 | 0.56±0.01 | 0.44±0.13 | 0.43±0.03 | 0.45±0.03 | 0.36±0.03 | 0.18±0.01 | 0.18±0.01 | 0.18±0.01 | -0.25±0.01 |
| **FD_Auto** | 0.14±0.00 | 1 | -0.12±0.02 | 0.38±0.02 | 0.31±0.02 | 0.06±0.03 | 0.12±0.09 | 0.66±0.01 | 0.11±0.01 | 0.37±0.01 | 0.36±0.02 | 0.37±0.02 | 0.34±0.01 | 0.32±0.03 |
| **FD_Skewness** | 0.21±0.01 | -0.01±0.00 | 1 | 0.46±0.01 | 0.57±0.03 | 0.68±0.07 | 0.26±0.03 | 0.24±0.03 | 0.35±0.03 | 0.34±0.01 | 0.32±0.01 | 0.42±0.03 | 0.41±0.02 | -0.37±0.04 |
| **FD_ABS** | 0.08±0.00 | 0.26±0.01 | 0.05±0.00 | 1 | 0.49±0.01 | 0.19±0.00 | 0.22±0.10 | 0.58±0.17 | 0.17±0.00 | 0.30±0.01 | 0.27±0.02 | -0.53±0.04 | -0.46±0.02 | 0.32±0.02 |
| **FD_ROC_res** | 0.07±0.00 | 0.19±0.01 | 0.04±0.02 | 0.41±0.10 | 1 | 0.49±0.01 | 0.86±0.15 | -0.48±0.03 | 0.41±0.01 | 0.18±0.01 | 0.18±0.02 | -0.20±0.02 | 0.37±0.03 | 0.04±0.01 |
| **FD_ROC_rec** | 0.08±0.00 | 0.03±0.01 | 0.05±0.01 | 0.05±0.01 | 0.38±0.01 | 1 | 0.96±0.17 | 0.51±0.04 | 0.38±0.01 | 0.15±0.02 | 0.21±0.01 | 0.00±0.03 | -0.09±0.01 | 0.10±0.01 |
| **FD_ABC** | 0.01±0.02 | 0.10±0.03 | 0.01±0.01 | 0.08±0.01 | 0.55±0.01 | 0.69±0.01 | 1 | -0.11±0.08 | 0.08±0.01 | 0.13±0.03 | 0.15±0.01 | 0.09±0.01 | 0.10±0.01 | -0.12±0.01 |
| **BW_Lnvar** | 0.04±0.01 | 0.05±0.00 | 0.02±0.01 | 0.00±0.00 | -0.03±0.01 | 0.03±0.01 | -0.01±0.02 | 1 | 0.17±0.01 | 0.00±0.01 | 0.58±0.02 | 0.66±0.06 | 0.62±0.04 | 0.36±0.02 |
| **BW_Auto** | 0.06±0.00 | 0.00±0.00 | 0.04±0.01 | 0.02±0.01 | 0.14±0.00 | 0.12±0.00 | 0.07±0.02 | 0.03±0.00 | 1 | 0.62±0.03 | -0.14±0.01 | 0.42±0.04 | 0.38±0.03 | -0.04±0.03 |
| **BW_Skewness** | 0.04±0.02 | 0.05±0.01 | 0.06±0.01 | 0.04±0.00 | 0.07±0.01 | 0.03±0.01 | 0.01±0.01 | 0.00±0.01 | 0.02±0.01 | 1 | 0.57±0.02 | 0.69±0.05 | 0.65±0.04 | -0.16±0.03 |
| **BW_ABS** | 0.02±0.01 | 0.24±0.01 | 0.08±0.01 | 0.23±0.01 | 0.14±0.01 | 0.15±0.00 | 0.00±0.01 | 0.05±0.00 | -0.03±0.01 | 0.05±0.00 | 1 | 0.05±0.01 | 0.10±0.01 | 0.21±0.01 |
| **BW_ROC_res** | 0.16±0.00 | 0.25±0.02 | 0.05±0.01 | -0.14±0.00 | -0.15±0.01 | 0.00±0.01 | 0.06±0.01 | 0.07±0.00 | 0.17±0.01 | 0.07±0.00 | 0.00±0.00 | 1 | 0.64±0.06 | 0.88±0.09 |
| **BW_ROC_rec** | 0.15±0.00 | 0.23±0.01 | 0.05±0.01 | -0.15±0.00 | 0.30±0.00 | -0.07±0.00 | 0.02±0.00 | 0.06±0.00 | 0.16±0.01 | 0.06±0.01 | 0.08±0.01 | 0.57±0.00 | 1 | 0.96±0.09 |
| **BW_ABC** | -0.05±0.02 | 0.08±0.02 | -0.07±0.01 | 0.12±0.00 | 0.20±0.00 | 0.11±0.00 | -0.02±0.01 | 0.07±0.00 | 0.09±0.00 | -0.16±0.01 | 0.04±0.00 | 0.25±0.00 | 0.72±0.04 | 1 |

Abbreviations, FD=fibre diameter, BW=body weight, Lnvar=natural log variance of the deviation, Auto=lag1 autocorrelation of the deviation, Skewness= skewness of the deviation, ABS=absolute change in the deviation, ROC_res=rate of change during the response phase of the weaning challenge, ROC_rec= rate of change during the recovery phase of the weaning challenge, ABC=area between curve during the weaning challenge.
