# Supplementary material for: Estimating the genetic parameters of resilience toward known and unknown disturbances in sheep using wool fibre diameter and body weight variability
Source: Genet Sel Evol. 2025 Jul 14;57:38. doi: 10.1186/s12711-025-00983-1 (PMC12257680; doi:10.1186/s12711-025-00983-1)
Supplement: Supplementary file 3 — Additional file 3: Table S3. Phenotypic correlations (±SE) between resilience indicators derived from standardised wool fibre diameter and body weight and health traits in sheep. [file 12711_2025_983_MOESM3_ESM.docx]

Additional file 3, Table S3

Phenotypic correlations (±SE) between resilience indicators derived from standardised wool fibre diameter and body weight and health traits in sheep.

| **Trait** | **DERM** | **DAG** | **BRWR** | **FLROT** | **CS** | **WEC** |
| --- | --- | --- | --- | --- | --- | --- |
| **FD_Lnvar** | 0.01 ±0.02 | -0.02 ±0.02 | 0.01 ±0.04 | 0.05 ±0.02 | -0.01 ±0.02 | -0.02 ±0.02 |
| **FD_Auto** | 0.01 ±0.00 | 0.04 ±0.01 | 0.03 ±0.00 | 0.01 ±0.01 | 0.01 ±0.00 | -0.01 ±0.00 |
| **FD_Skewness** | 0.01 ±0.00 | 0.01 ±0.02 | 0.01 ±0.02 | 0.02 ±0.02 | 0.03 ±0.02 | -0.05 ±0.02 |
| **FD_ABS** | 0.03 ±0.00 | 0.02 ±0.01 | 0.01 ±0.02 | 0.03 ±0.01 | 0.02 ±0.02 | -0.01 ±0.01 |
| **FD_ROC_resp** | -0.01 ±0.02 | -0.01 ±0.00 | -0.00 ±0.01 | -0.00 ±0.01 | -0.01 ±0.02 | -0.01 ±0.02 |
| **FD_ROC_reco** | -0.00 ±0.02 | -0.00 ±0.01 | -0.01 ±0.01 | 0.01 ±0.00 | -0.01 ±0.02 | -0.01 ±0.00 |
| **FD_ABC** | 0.01 ±0.01 | -0.01 ±0.02 | -0.04 ±0.04 | -0.00 ±0.02 | -0.01 ±0.02 | -0.02 ±0.01 |
| **BW_Lnvar** | 0.01 ±0.02 | 0.04 ±0.02 | 0.01 ±0.02 | 0.01 ±0.02 | -0.03 ±0.01 | -0.01 ±0.01 |
| **BW_Auto** | -0.01 ±0.00 | 0.00 ±0.00 | -0.02 ±0.01 | 0.03 ±0.01 | 0.01 ±0.00 | 0.02 ±0.01 |
| **BW_Skewness** | 0.02 ±0.01 | 0.00 ±0.00 | 0.01 ±0.01 | 0.04 ±0.02 | -0.06 ±0.01 | 0.04 ±0.02 |
| **BW_ABS** | 0.04 ±0.01 | -0.03 ±0.02 | 0.03 ±0.01 | 0.03 ±0.02 | 0.07 ±0.01 | 0.02 ±0.01 |
| **BW_ROC_resp** | 0.00 ±0.00 | 0.01 ±0.00 | 0.00 ±0.00 | 0.02 ±0.01 | 0.02 ±0.01 | -0.00 ±0.00 |
| **BW_ROC_rec** | 0.00 ±0.00 | -0.01 ±0.01 | -0.00 ±0.00 | 0.01 ±0.00 | 0.04 ±0.02 | -0.00 ±0.00 |
| **BW_ABC** | 0.01 ±0.01 | -0.02 ±0.03 | -0.01 ±0.01 | 0.03 ±0.01 | 0.05 ±0.02 | 0.04 ±0.01 |
| Abbreviations, FD=fibre diameter, BW=body weight, Lnvar=natural log variance of the deviation, Auto=lag1 autocorrelation of the deviation, Skew= skewness of the deviation, ABS=absolute change in the deviation, ROC_resp=rate of change during the response phase of the weaning challenge, ROC_reco= rate of change during the recovery phase of the weaning challenge, ABC=area between curve during the weaning challenge, DERM = wool dermophilisis, DAG = dag score, BRBW = breech wrinkle, FLROT = fleece rot score, CS = body condition score, WEC = worm egg count. | | | | | | |
